# Supplementary material for: The influence of shot noise on the performance of phase singularity-based refractometric sensors
Source: Nanophotonics. 2025 Jun 19;14(14):2463–72. doi: 10.1515/nanoph-2025-0101 (PMC12273541; doi:10.1515/nanoph-2025-0101)
Supplement: Supplementary file 1 — Supplementary Material Details [file j_nanoph-2025-0101_suppl_001.pdf]

# Supporting Information for ”The influence of shot noise on the performance of phase singularity-based refractometric sensors”

Valeria Maslova,[1] Georgy Ermolaev,[2] Evgeny S. Andrianov,[3,1]
Aleksey V. Arsenin,[2] Valentyn S. Volkov,[2] and Denis G. Baranov[1,*]

[1]*Moscow Center for Advanced Studies, Moscow, 123592, Russia*

[2]*Emerging Technologies Research Center, XPANCEO, Internet City, Emmay Tower, Dubai, United Arab Emirates*

[3]*285011 Dukhov Research Institute of Automatics (VNIIA) , 22 Sushchevskaya, Moscow 127055, Russia*

## CONTENTS

S1. Reflection amplitudes 2

S2. Mean value of maximum phase change 3

S3. Standard deviation of maximum phase change 4

S4. Probability distribution of the maximum phase change 5

S5. Comparison of sensor resolution determined by noise and sensitivity 11

* baranov.mipt@gmail.com

## S1. REFLECTION AMPLITUDES

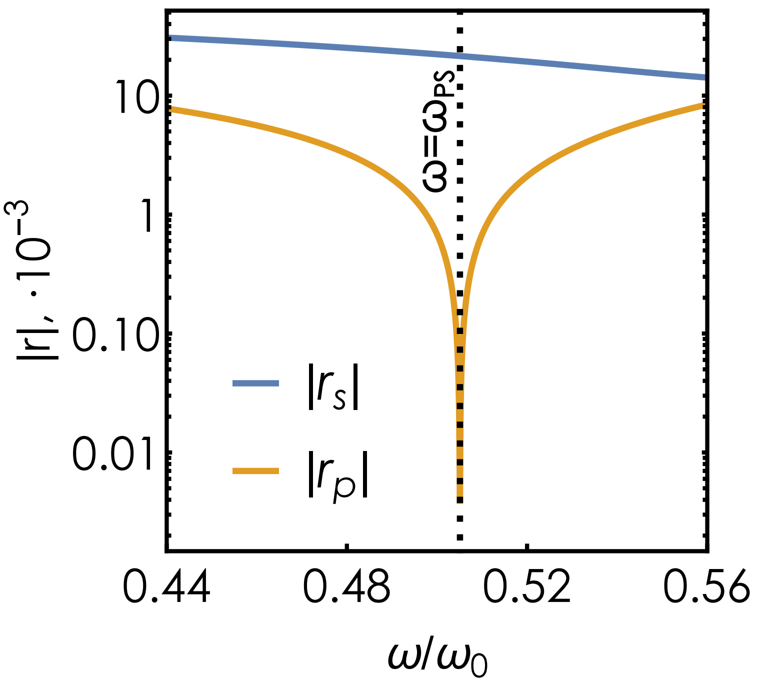


FIG. S1. Behavior of the reflection amplitudes near the phase singularity frequency at $\theta = \theta_{PS}$. Reflection function $|r_p|$ turns to zero at $\omega = \omega_{PS}$ while $|r_s|$ remains nonzero but quit small due to close impedance values of the analyte and the uniaxial material for our model system.

## S2. MEAN VALUE OF MAXIMUM PHASE CHANGE

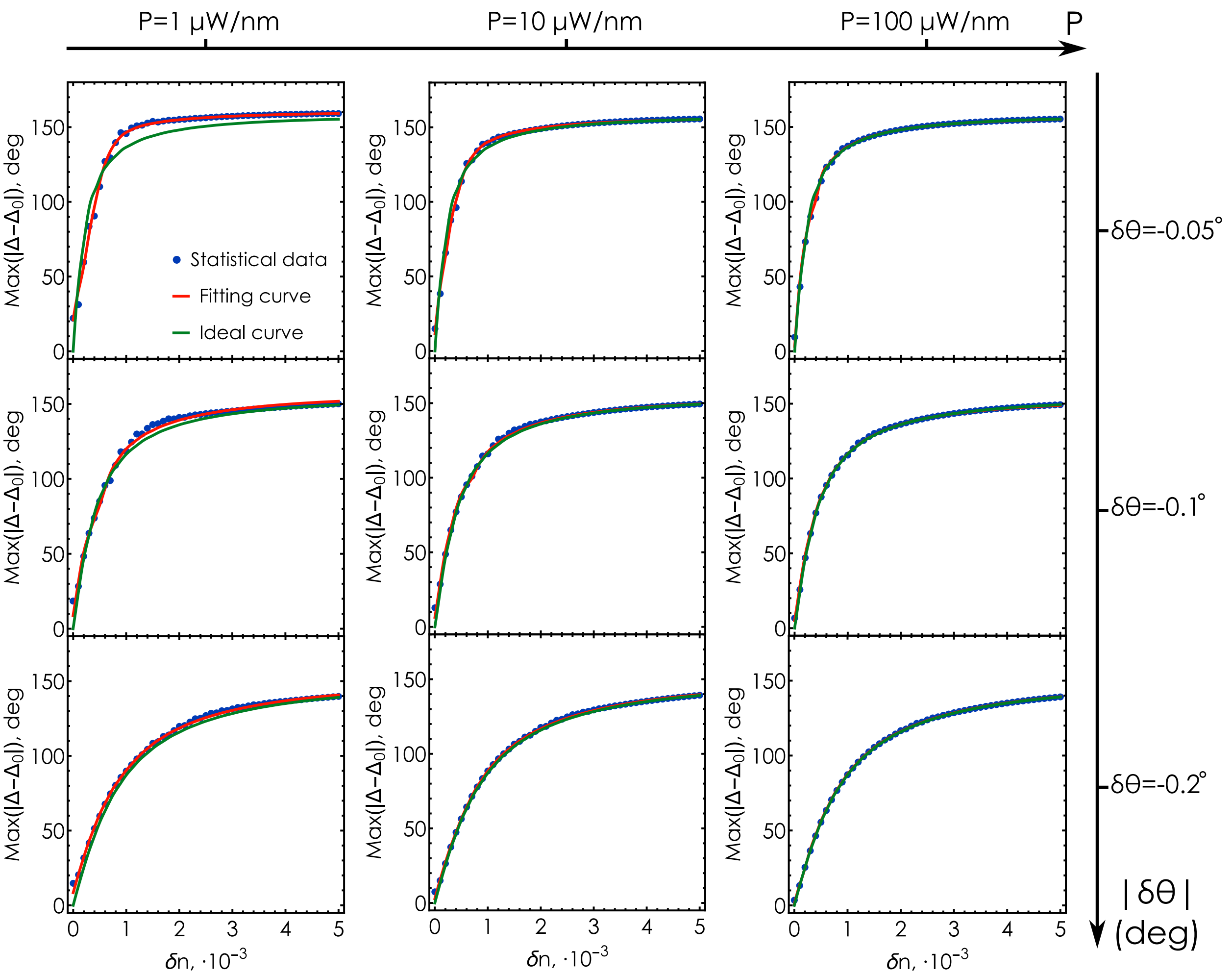


FIG. S2. Behavior of mean values of maximum phase changes $\mu(\delta n)$ for a set of refractive index changes with increasing spectral power $P$ (from left to right) and increasing deviation $|\delta\theta|$ of the incidence angle $\theta = \theta_{PS} + \delta\theta$ from the angle of phase singularity $\theta_{PS}$ (from top to bottom) obtained from statistical data in comparison with fitting and ideal sensitivity curves.

## S3. STANDARD DEVIATION OF MAXIMUM PHASE CHANGE

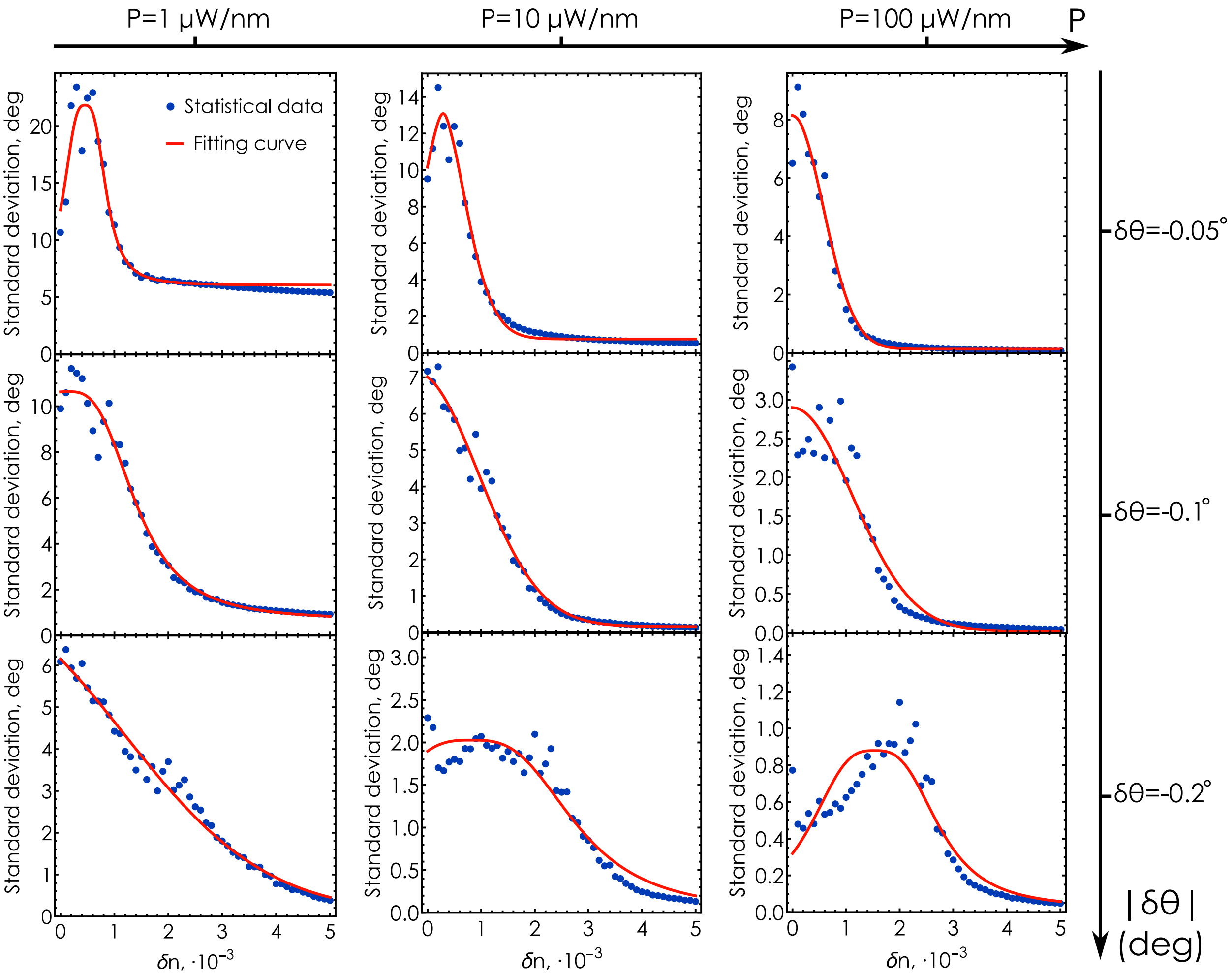


FIG. S3. Behavior of standard deviations of maximum phase changes for a set of refractive index changes with increasing spectral power $P$ (from left to right) and increasing deviation $|\delta\theta|$ of the incidence angle $\theta = \theta_{PS} + \delta\theta$ from the angle of phase singularity $\theta_{PS}$ (from top to bottom) obtained from statistical data in comparison with fitting and ideal sensitivity curves.

## S4. PROBABILITY DISTRIBUTION OF THE MAXIMUM PHASE CHANGE

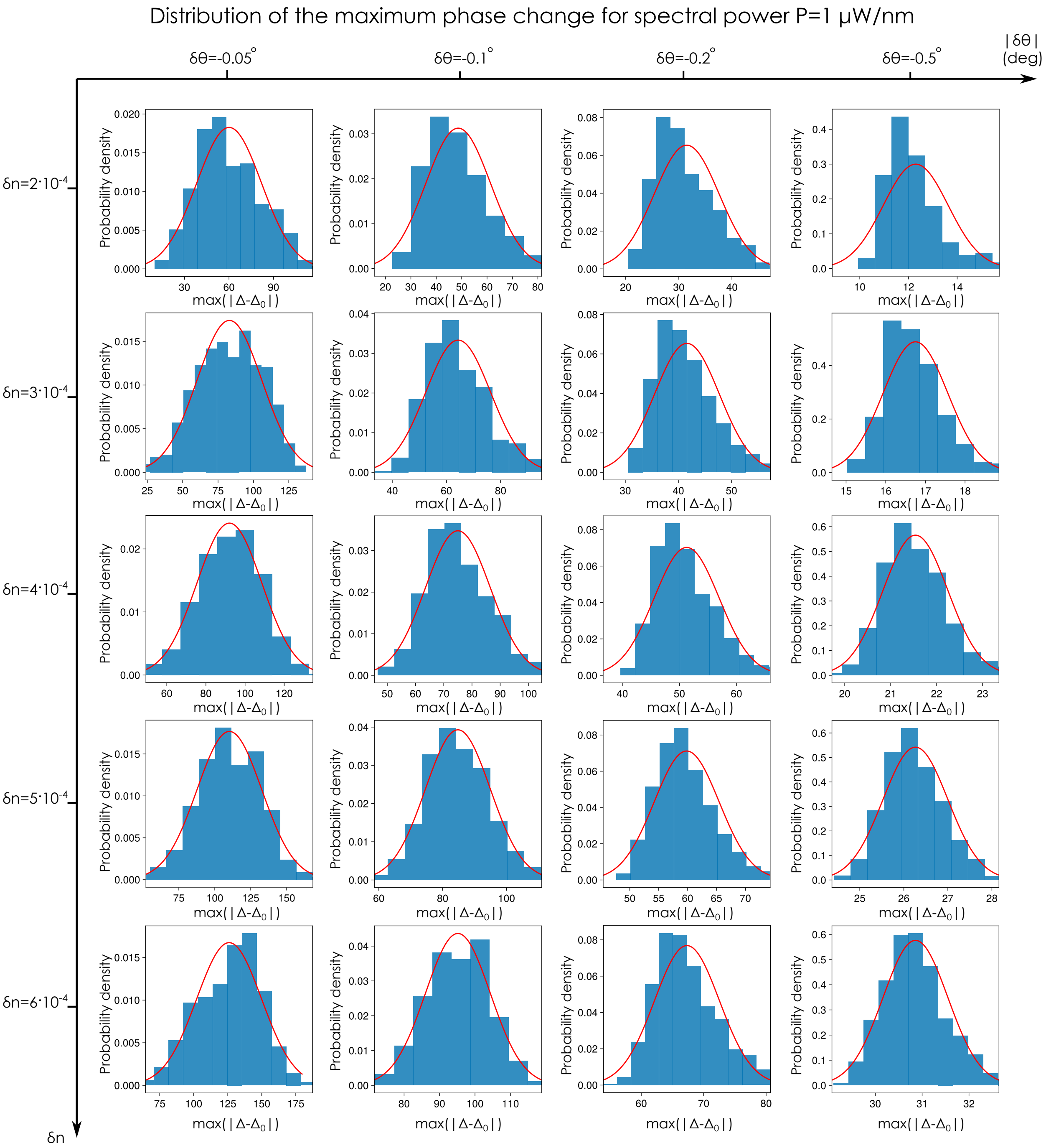


FIG. S4. Histograms of probability distributions of maximum phase change for a set of refractive index changes $\delta n$ and deviation angles $\delta\theta$ for spectral power of $P = 1\,\mu\mathrm{W/nm}$. Red curves indicate fitting of statistical data with normal distribution.

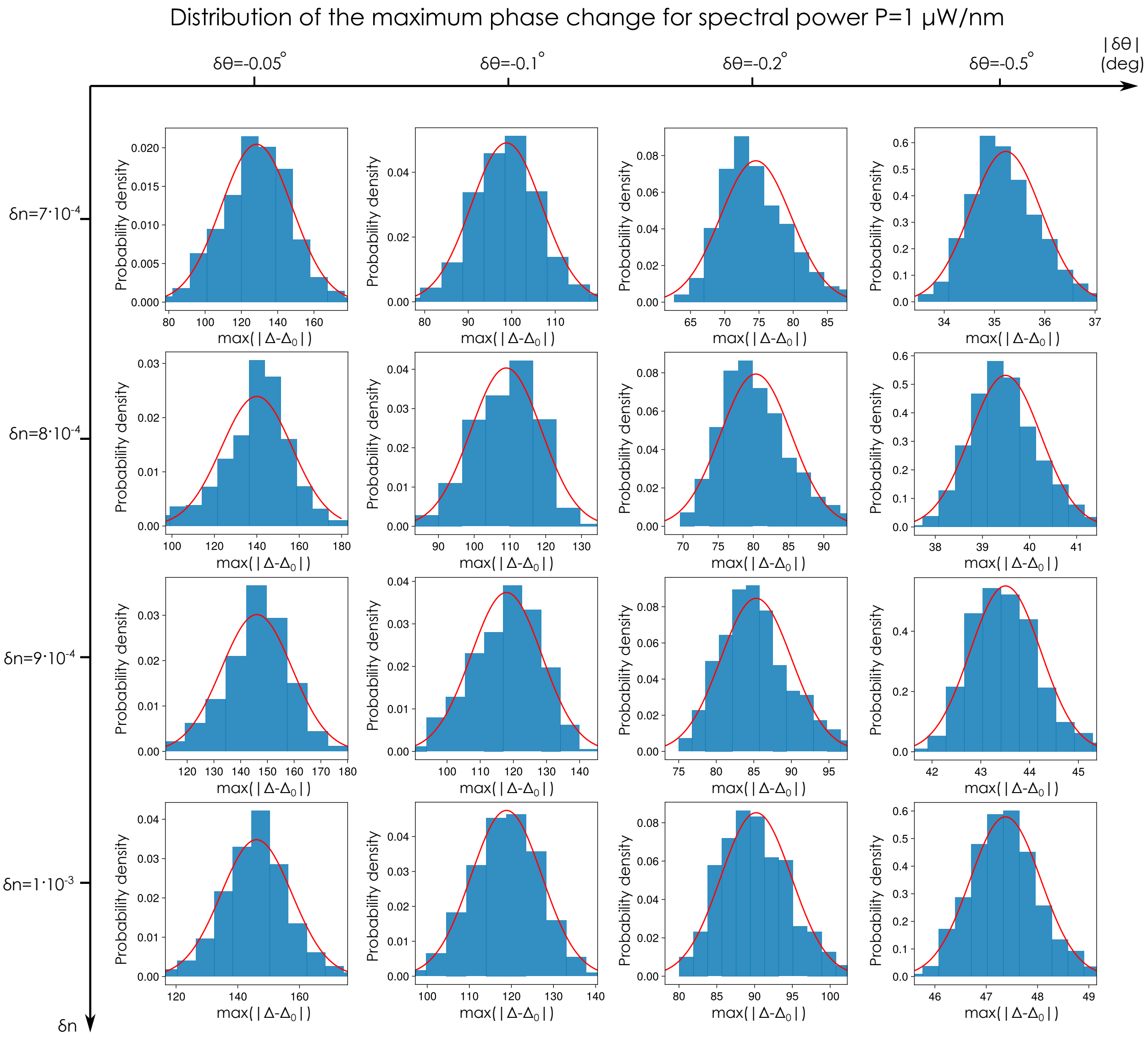


FIG. S5. Histograms of probability distributions of maximum phase change for a set of refractive index changes $\delta n$ and deviation angles $\delta\theta$ for spectral power of $P = 1\,\mu$W/nm. Red curves indicate fitting of statistical data with normal distribution.

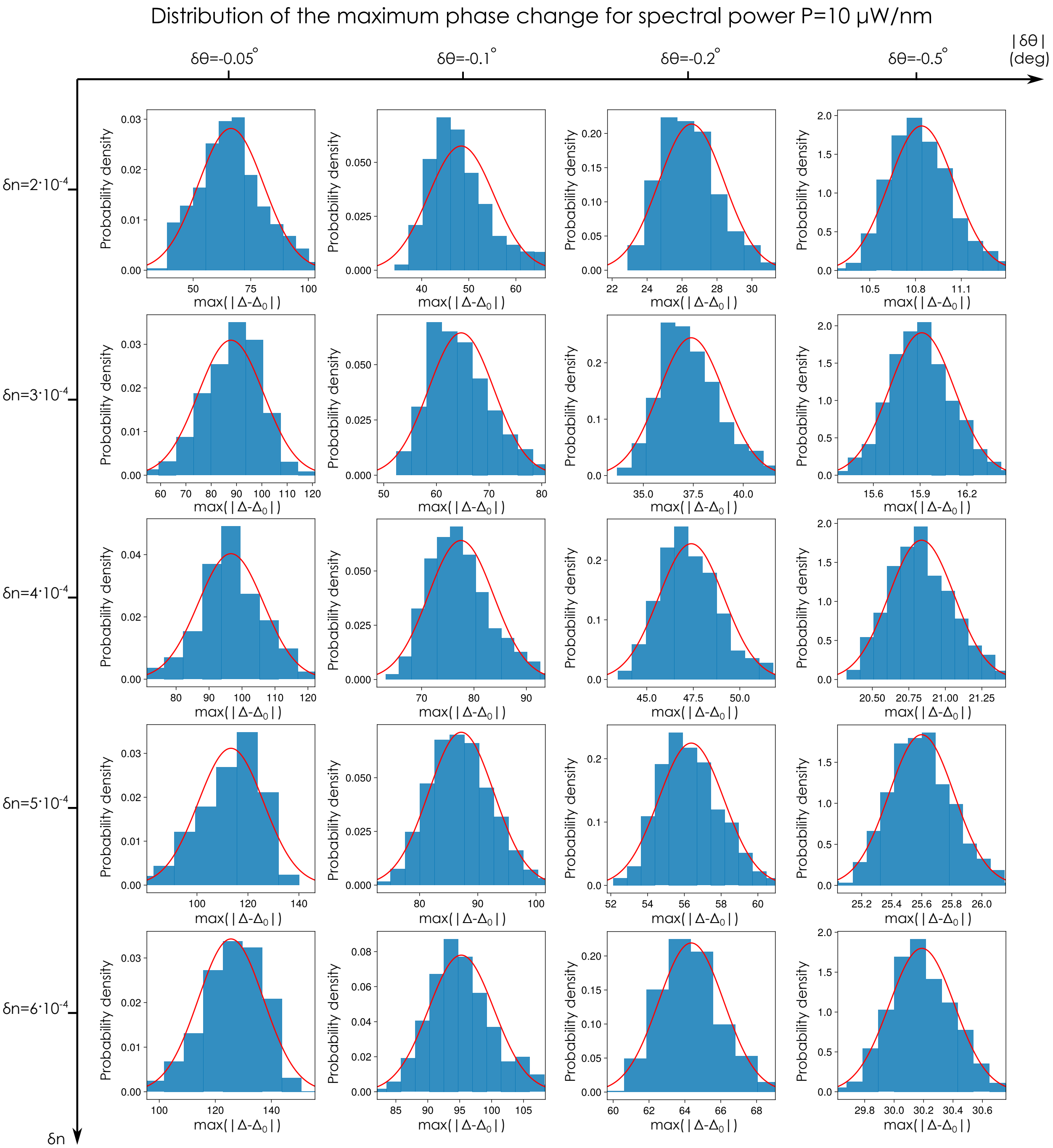


FIG. S6. Histograms of probability distributions of maximum phase change for a set of refractive index changes $\delta n$ and deviation angles $\delta\theta$ for spectral power of $P = 10\,\mu\mathrm{W/nm}$. Red curves indicate fitting of statistical data with normal distribution.

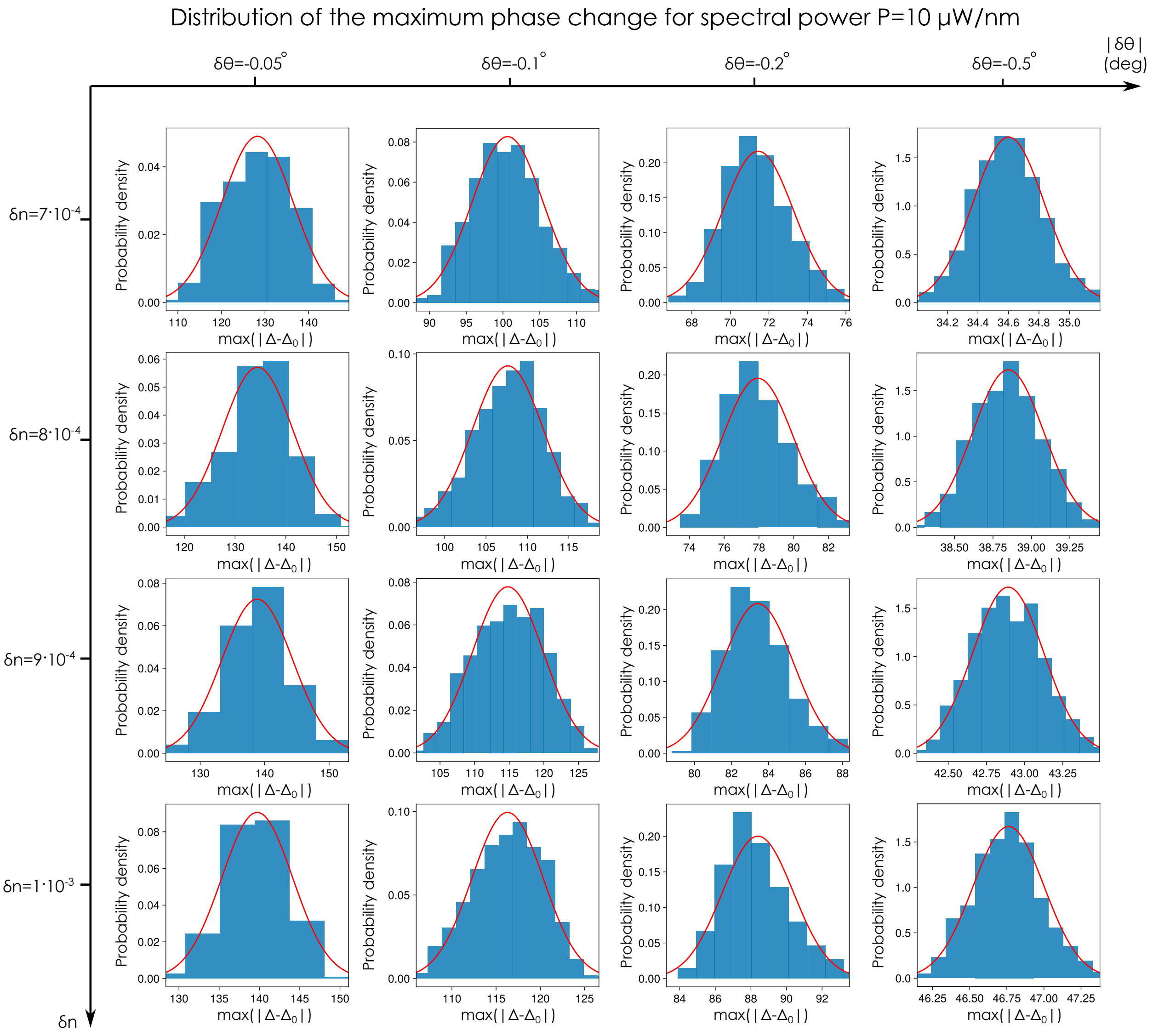


FIG. S7. Histograms of probability distributions of maximum phase change for a set of refractive index changes $\delta n$ and deviation angles $\delta\theta$ for spectral power of $P = 10\,\mu\mathrm{W/nm}$. Red curves indicate fitting of statistical data with normal distribution.

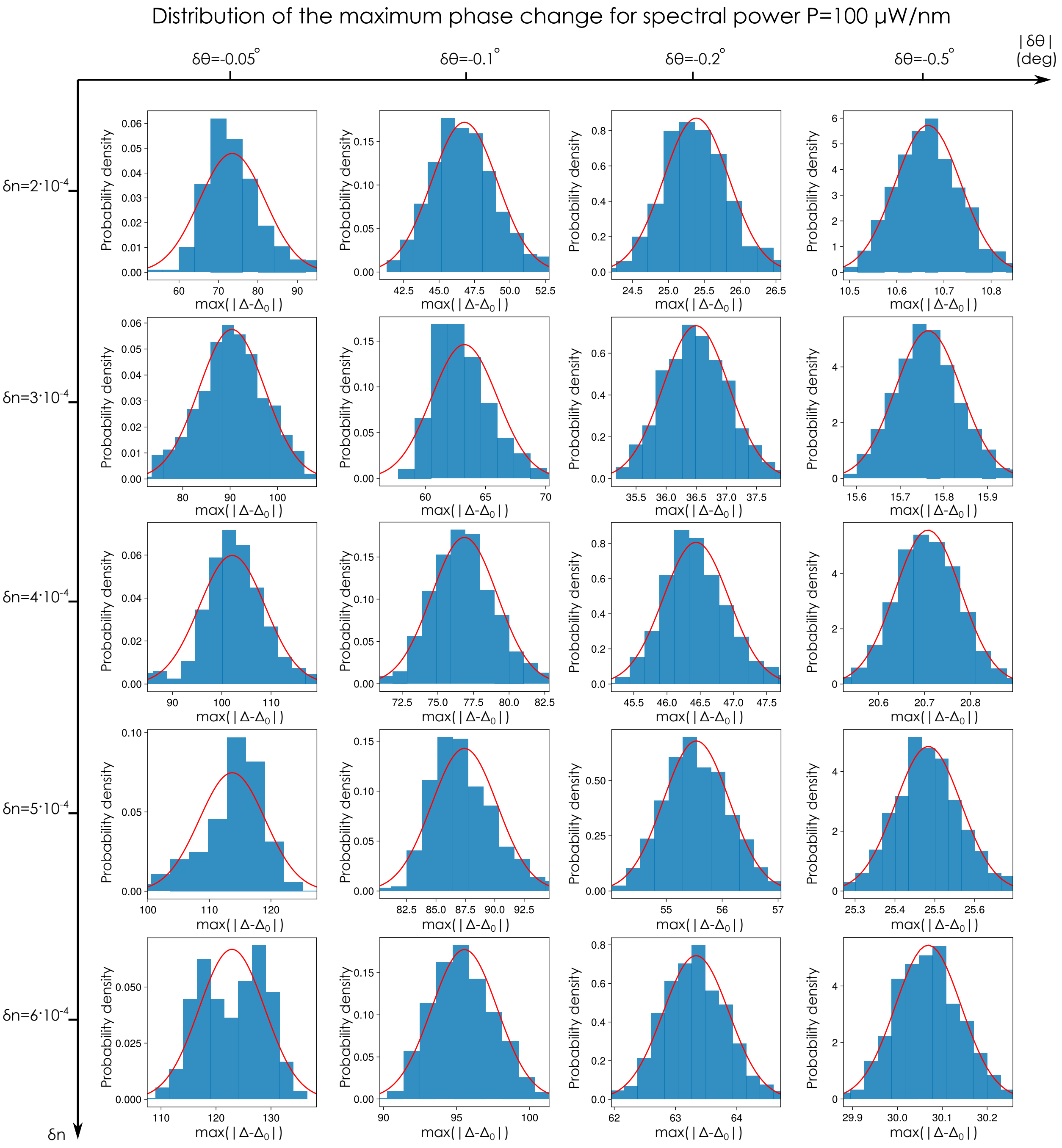


FIG. S8. Histograms of probability distributions of maximum phase change for a set of refractive index changes $\delta n$ and deviation angles $\delta\theta$ for spectral power of $P = 100\,\mu\mathrm{W/nm}$. Red curves indicate fitting of statistical data with normal distribution.

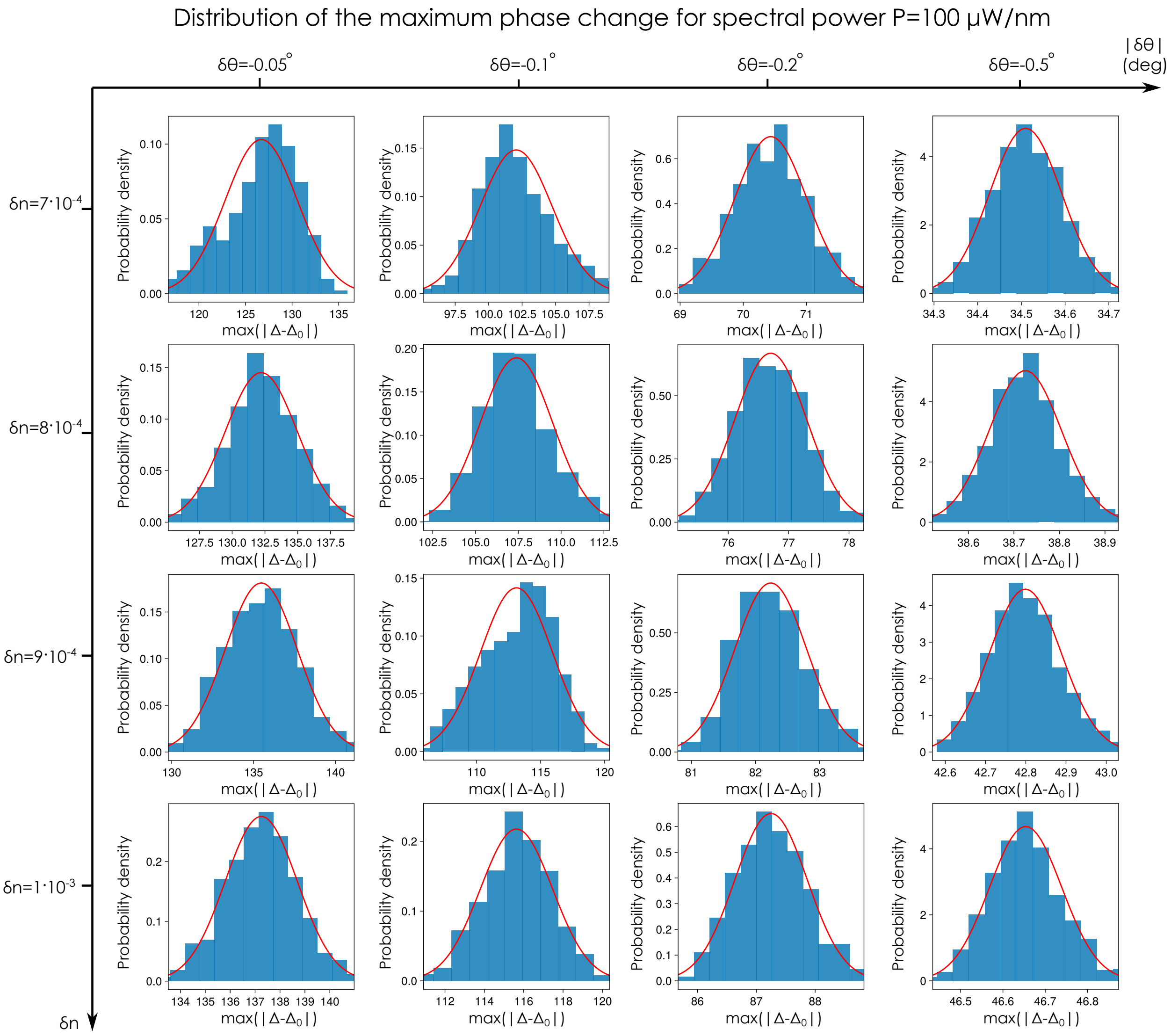


FIG. S9. Histograms of probability distributions of maximum phase change for a set of refractive index changes $\delta n$ and deviation angles $\delta\theta$ for spectral power of $P = 100\,\mu\mathrm{W/nm}$. Red curves indicate fitting of statistical data with normal distribution.

## S5. COMPARISON OF SENSOR RESOLUTION DETERMINED BY NOISE AND SENSITIVITY

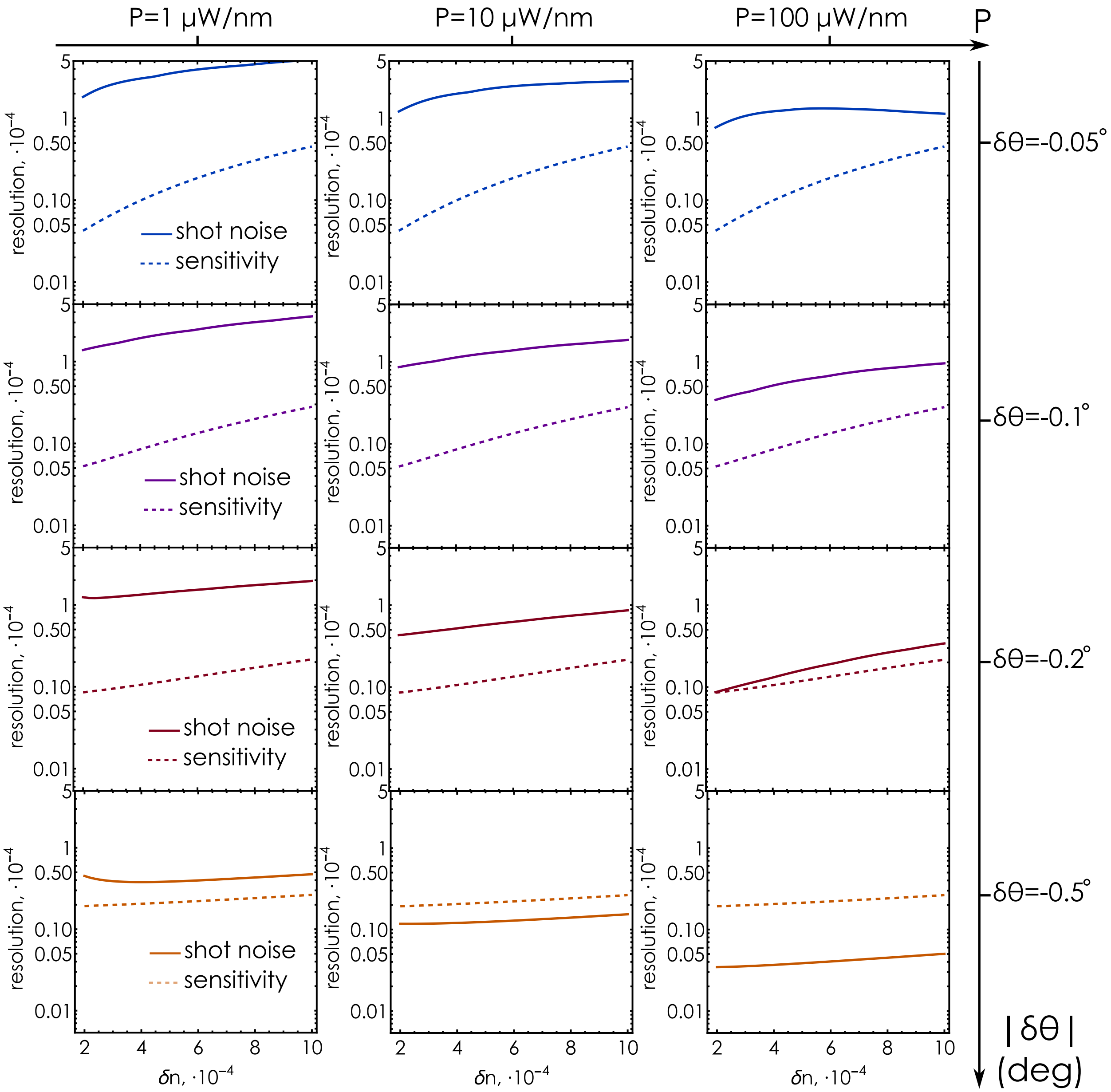


FIG. S10. Sensor resolution determined by shot noise (solid line) versus resolution determined by sensitivity (dashed line) for a set of deviation angles and source spectral powers.
